# Are women adequately informed about the use of instrumentation during vaginal delivery? A prospective review of the information on instrumental delivery provided to pregnant women and a retrospective review of the quality of consent for instrumental delivery

**DOI:** 10.1111/codi.70050

**Published:** 2025-03-11

**Authors:** Alessandra Orlando, Gregory P. Thomas, Ruwan Fernando, Jamie Murphy, Nada Elsaid, Stella Dilke, Carolynne J. Vaizey

**Affiliations:** ^1^ Sir Alan Parks Department of Physiology St Mark's Hospital, National Bowel Hospital London UK; ^2^ Imperial College London London UK; ^3^ Imperial NHS Healthcare London UK; ^4^ Cleveland Clinic London UK

**Keywords:** anal sphincter injuries, assisted delivery, consent process, faecal incontinence, instrumental delivery, OASIS, women's knowledge

## Abstract

**Aim:**

Instrumental delivery typically describes the use of ventouse or forceps to aid vaginal delivery. They are used in 10%–15% of all vaginal deliveries and in almost a third of all primiparous deliveries. They are associated with an increased risk of maternal and neonatal injury. Such maternal injuries may lead to significant functional problems such as faecal incontinence and these conditions can be life changing. Informed consent should be obtained before any medical procedure. This should be taken well before the procedure, documented clearly, and alternatives should be discussed. The aim of this study was to assess the information provided to pregnant women and the quality of consent obtained prior to instrumental vaginal delivery.

**Method:**

Patients were approached at 36 weeks’ gestation, prior to a planned vaginal delivery. The study was undertaken in the Obstetric Department of a busy District General Hospital in the UK. The patients’ understanding of labour, vaginal delivery and the use of instrumentation was assessed. Clinical notes of women who had an assisted vaginal delivery were reviewed to ascertain time of consent for use of instrumentation and amount of analgesia received at that point.

**Results:**

A total of 138 patients were included for prospective assessment. Only 4% were able to describe all stages of labour, 17% could describe some of the stages of labour and 78% were unable to describe any of the stages of labour. Eighty per cent of the participants were unaware that instrumentation may be used during delivery, 21% were aware that it may be used if necessary and only 2% were aware of the risk of maternal injury. Fifty‐nine case notes were reviewed. All had undergone either forceps or ventouse instrumentation. Eighteen per cent showed no record of informed consent. Sixty‐one per cent showed evidence of verbal consent and 20% had a signed consent in the clinical notes. All had been consented for use of instrumentation during the second stage of labour, often when opioid analgesia had been administered prior to consent.

**Conclusion:**

Knowledge about labour and the use of instrumentation during delivery appeared to be poor. The quality of consent taken for instrumental delivery appeared to be sub‐standard. Larger studies, done post the COVID‐19 epidemic, are needed to confirm that similar practices still occur in other hospitals and, if this is the case, urgent measures need to be taken to correct this.

**What does this paper add to the literature?**

Instrumental delivery is a risk factor for anal sphincter injury and lifelong faecal incontinence. To the best of our knowledge, this is the first study to assess women's knowledge about instrumental delivery and its potential consequences in late pregnancy and also to look at the consent processes for assisted delivery.

## INTRODUCTION

In the UK, forceps and ventouse assisted (instrumental) delivery are used in 10%–15% of all deliveries [1] and in 23% of primiparous deliveries [2]. The use of instrumentation is associated with an increased risk of maternal and neonatal injury [3, 4, 5, 6, 7, 8, 9]. Pelvic floor injuries consequent to instrumental vaginal delivery may lead to significant functional problems, such as faecal incontinence, perineal pain, dyspareunia, bladder dysfunction and bowel evacuatory problems.

The Green Top guidelines of the Royal College of Obstetricians and Gynaecologists (RCOG) state that ‘women should be informed about assisted vaginal birth in the antenatal period, especially during their first pregnancy. If they indicate specific restrictions or preferences then this should be explored with experienced obstetricians, ideally in advance of labour’ and ‘When midpelvic or rotational birth is indicated, the risks and benefits of assisted vaginal birth should be compared with the risks and benefits of second stage caesarean birth for the given circumstances and skills of the operator’ [1].

This study has two parts. The first was to determine the level of understanding amongst pregnant women about the different stages of labour and the potential use of instrumentation. The second was to determine the quality of consent obtained when instrumentation was used.

The study is based on the results presented in Chapters 8 and 10 from the MD thesis ‘The impact of obstetric anal sphincter injuries’ [10] deposited in July 2024 at Imperial College London by Alessandra Orlando, member of the author list for this article.

## METHOD

Ethical approval was obtained from the West Midlands South Birmingham Research Ethics Committee (Iras number 289693) in December 2020. The study was conducted at London North West University Healthcare Trust (LNWH) (Northwick Park Hospital [NPH] and St Mark's Hospital). NPH is a large District General Hospital where around 4200 births take place each year [11] and St Mark's Hospital is a tertiary referral centre for pelvic floor disorders such as anal and faecal incontinence after obstetric anal sphincter injury. Participants were identified from the antenatal care database at NPH.

**FIGURE 1:**
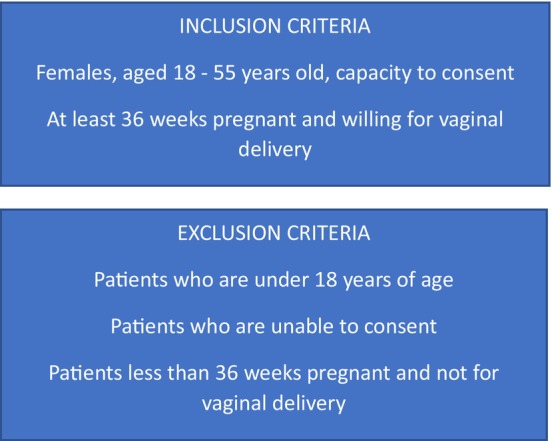
Inclusion and exclusion criteria.

For the first part of the study, women aged between 18 and 55 years old, who were at least 36 weeks pregnant and planning to have a vaginal delivery, were prospectively recruited between 2 July 2021 and 30 June 2022 (Figure 1). Once identified, they were contacted via telephone to further assess their capacity, ability to speak and understand English, and eligibility. The participants who agreed to take part in the study were consented and interviewed via telephone.

The questionnaire had six questions.

1. Is this your first pregnancy?
2. What do you know about labour?
3. Have the stages of labour been explained to you? If yes, can you explain them to me in your own words?
4. Have you attended antenatal classes or met a midwife or an obstetrician at this stage?
5. Have they mentioned instrumentation? If yes, what have they told you about that?
6. What do you know about it?

Answers were analysed in a qualitative and quantitative manner. Descriptive analysis was used for quantitative analysis of the data. Qualitative thematic analysis of the open‐ended questions in the questionnaire was applied following the method described by Braun and Clarke [12]. Themes and patterns were identified with an inductive approach. Data were read and reread before coding and identifying themes, which were then discussed and agreed with all authors.

A group of clinical experts (colorectal surgeons with special interest in the pelvic floor, obstetricians with special interest in urogynaecology, pelvic floor physiotherapists) were involved with the study design.

The study was initially presented at a general patient forum meeting at LNWH. Following this, the questionnaire was presented to a focus group of women who had previously received instrumentation at childbirth. These women voluntarily took part in the focus group; they were recruited through Mush, a social media platform for expectant and new mothers. The questions were modified according to their feedback in relation to the language used and the number of questions in relation to the sensitive topic treated in the research.

If the questioning caused anxiety or curiosity about an instrumental delivery, patients were directed to the local obstetric team for further information.

For the other (non‐consecutive) part of the study, the clinical notes were reviewed of patients who had an instrumental vaginal delivery at NPH between 2 December 2020 and 27 January 2021. The consent process was interrogated to ascertain which risks were explained in relation to the use of instrumentation, when the women were consented and what level of analgesia (pain killers) had been given at the time of consent.

## RESULTS

### Prospective assessment of information provided to pregnant patients

A total of 595 women were eligible and were contacted. Of these, 138 agreed to complete the questionnaire. Fifty‐nine participants (43%) were in their first pregnancy (Table 1).

**TABLE 1:** Parity of participants.

Pregnancy	Number of participants (%)
First	59 (42.7)
Second	50 (36.2)
Third	17 (12.3)
Fourth	5 (3.6)
Fifth	3 (2.2)
Sixth	2 (1.4)
Seventh	0
Eighth	2 (1.4)

The framework analysis of the answers to open‐ended questions highlighted different themes.

### First question ‘What do you know about labour?’

From the online National Health Service (NHS) data dictionary [13], the definition of labour is ‘Labour is a period of time when there are painful contractions and changes to the cervix that result in the birth of a baby and end with the expulsion of the placenta and membranes.’

Framework analysis of the answers to this question highlighted the themes given in Table 2.

**TABLE 2:** Themes highlighted by framework analysis of answers to ‘What do you know about labour?’.

Themes	Quoted answer
Ignorance (doesn't know what labour is)	I don't know
Inability to explain, feels unsure	I am not quite sure how to explain
Minimizing/simplifying when replying with a simple and reassuring comment	I was told by the midwife that everything is ok
Pain	I only know it's a lot of pain
Replying with a single piece of information or instruction received by the health professional	The midwife explained about that, how it will start, then wait for regular contractions and then go to hospital
Participant replies explaining her previous personal experience of labour	I know everything about labour. First water breaks then contractions
Participant replies with a simple statement	Labour is when the baby comes out

Thirty‐five women (25.36%), all at their first or second pregnancy, gave a more accurate definition of labour, listing the main signs and symptoms (release of mucous plug, water break, starting of contractions and increasing frequency of contractions) as part of information received by midwives and doctors. This information was provided by health professionals to instruct women about when would be the proper time to attend hospital. The remaining 103 participants (74.64%) were not able to give a complete answer about labour (Table 2).

### Second question ‘Have stages of labour been explained you? If yes, can you explain to me in your own words?’

As explained in the NHS website there are three stages of labour [13]: ‘during the first stage of labour, contractions make your cervix gradually open (dilate). This is usually the longest stage of labour. At the start of labour, your cervix starts to soften so it can open. This is called the latent phase and you may feel irregular contractions. It can take many hours, or even days, before you're in established labour. Established labour is when your cervix has dilated to about 4 cm and regular contractions are opening your cervix. Your cervix needs to open about 10 cm for your baby to pass through it. This is what's called being fully dilated. When you reach the end of the first stage of labour, you may feel an urge to push. The second stage of labour lasts from when your cervix is fully dilated until the birth of your baby. The third stage of labour happens after your baby is born, when your womb contracts and the placenta comes out through your vagina.’

When asked about this, 84 (60.9%) participants out of the total of 138 stated that they had not been taught about the stages of labour. Fifty‐four (39.1%) stated that they had.

The 54 participants who answered that they knew about stages of labour were asked to explain them in their own words. Six participants (4.3% of the total) were able to give a complete explanation about labour and its stages. Twenty‐four participants (17.3% of the total) gave a partial explanation of stages of labour. They mentioned some of the stages or gave a partially correct answer (e.g., ‘if we are having back pain, cramps, dilation started but not much, early stage. I don't know what happens after: maybe pain increases’) showing that they do not know what stages of labour are. Ten participants (7.2% of the total) did not know how to explain (‘yes, three stages, I don't know how to explain’).

Fourteen participants (10.1% of the total) replied with an answer that was off topic (‘There are different kinds of labour, strong, quick, long’) showing that they really did not know what stages of labour are.

### Third question ‘Have you been offered and attended any antenatal classes?’

Out of a total of 138 participants, 115 participants (83.3%) did not attend antenatal classes because they were not offered any or were unaware of them.

Out of the total of 115 participants, eight explained that it was because of the COVID‐19 pandemic, three did not know what an antenatal class was and 20 had attended in the past, during a previous pregnancy.

Twenty‐four participants replied that they attended antenatal classes, three of whom through private antenatal classes; 11 did it online through a link provided by the hospital.

### Fourth question ‘Have you met a midwife and/or an obstetrician during your pregnancy?’

**FIGURE 2:**
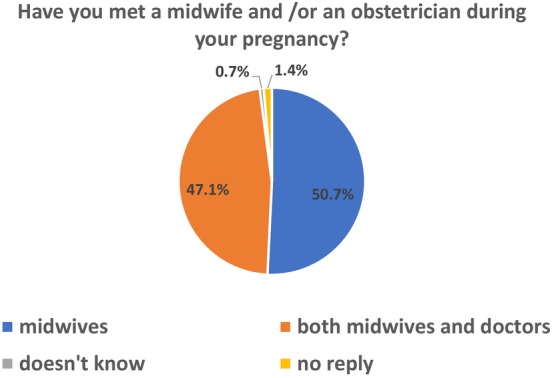
Have you met a midwife and/or an obstetrician during your pregnancy?

Seventy (50.7%) women were exclusively seen by midwives during their antenatal appointments and 65 women (47.1%) met both midwives and doctors (Figure 2). One participant (0.7%) did not know what to answer, two participants did not reply (1.4%).

### Fifth question ‘Have health professionals mentioned instrumentation (forceps and ventouse)?’

Ninety‐five participants (68.8%) were not informed about instrumentation; 15 (10.8%) did not know what instrumentation was. The answers of the 28 (20.3%) participants who replied ‘yes’ when asked ‘what have health professionals told you about that?’ are represented in Table 3.

**TABLE 3:** What have health professionals told you about instrumentation?

What have health professionals told you about instrumentation?	Number of participants (%)
It is used if needed	7 (6)
It is used to help the baby coming out	11 (9)
Knows about instrument from previous personal experience	3 (2)
Aware of risk of tear	3 (2.2)
Aware of risk of temporary mark or change in shape of the head of the baby following use of instrumentation	8 (7)

### Sixth question ‘What do you know about instrumentation?’

This was specifically asked to understand what the common knowledge is about instrumentation and its use, regardless of what the health professionals informed women about. Eighty participants (58%) replied that they did not know anything about instrumentation.

The remaining 58 participants (42%) gave an answer about their general knowledge about instrumentation. Framework analysis of the answers identified six main topics (Table 4).

**TABLE 4:** Framework analysis of the answers to the sixth question.

Topic	Some of the answers (quoted)
(1) Feeling scared about instrumentation	I think it is not a safe delivery In my country of origin (Romania) it is illegal to use forceps
(2) Instruments are used to help the baby to come out	They are used if the baby is stuck They are used if the mother has difficulties to push
(3) Only knows about their existence, but does not know anything more	All I know from the antenatal classes I attended for my first pregnancy is that there are forceps or vacuum that could be used during delivery. I don't know anything else I'm aware of these but nobody explained to me anything. I know these exist but I don't know anything more
(4) Aware of the existence of these instruments from their previous delivery or family or friends’ experience	I know my sister gave birth with forceps and it was horrible. I don't know anything more than this I didn't know what instrumentation was with my first child—they said they had to pull her out. After delivery I read about forceps and understood that complications can happen more often with the forceps, but I am not aware of risks for the mother
(5) Aware of related risks for baby and/or mother	I know there are some risks for the baby, not for the mum but I am not sure Forceps were used to deliver my friend's baby, not sure about details; baby had few bruises on her head; I think she had a lot of tearing, pretty bad
(6) Knowledge related only to their own research on the internet or on TV	I watched videos and saw the forceps and vacuum used I know instruments from the internet. They are used when baby needs help to come out. I know there are some risks for the baby, not for the mum but I am not sure

Twenty‐nine participants (21%) were aware that instruments are used to help the baby to come out. Only three participants (2.2%) were aware of a potential risk of tearing in relation to use of instrumentation.

### The second part: retrospective assessment of quality of consent for instrumentation

Of the 74 instrumental vaginal deliveries (either forceps or ventouse assisted) that took place at LNWH in the selected time frame, 59 case notes were available and were reviewed. In 18.6% (*n* = 11) of the case notes there was no record of informed consent. In 61% (*n* = 36) documentation of verbal consent was made in the notes and in 20.3% (*n* = 12) a separate signed consent form was part of the notes (Table 5).

**TABLE 5:** Type of consent obtained as recorded in clinical notes.

Status of consent in the clinical notes	Extracts from clinical notes	% (*n*)
Verbal consent documented	‘27/12/2020, time 6:10 am pushed for 1 h, feels tired, suspicious CTG; 6:25 verbal consent obtained for Kiwi delivery, 6:31 am Kiwi applied, 2 pulls and episiotomy, 6:35 am baby delivered’	61 (36)
Signed consent form one	Main risks: bleeding infection clots (lungs/legs), damage to bladder, bowel, vessels, ureters, baby, perineal 3rd/4th tears, urinary/bowel incontinence, placenta accreta/previa	20.3 (12)
No record of informed consent	‘20/1/2021, time 21:53 patient exhausted and uncomfortable with VE, decision made for Kiwi, 2 pulls and episiotomy’ ‘20/01/2021, 12:45 decelerations, decision to pull out with forceps in room’	18.6 (11)

Abbreviations: CTG, cardiotocography; VE, vaginal examination.

When consent was documented, this was obtained during the second stage of labour when opioid analgesia had already been administered.

Analgesia provided during labour by the time the consent was taken was an epidural infusion of bupivacaine 0.1% w/v and fentanyl 2 μg/mL, pethidine 100 mg intramuscularly and nitrous oxide. At the time of consent, the recorded amount of analgesia given was as follows: 57% (*n* = 34) of participants received an epidural, on average four boluses +/− 2.4 SD (maximum 11 boluses, minimum 1 bolus), 18% (*n* = 11) received pethidine 100 mg intramuscularly at least once during labour and 45.7% (*n* = 27) had nitrous oxide.

## DISCUSSION

At LNWH approximately 350 deliveries take place every month; around 50 of these are instrumental deliveries, that is, around one in seven or 14%.

The Green Top guidelines NO26 [14] of the Royal College of Obstetricians and Gynaecologists state that ‘Women should be informed about assisted vaginal birth in the antenatal period, especially during their first pregnancy. If they indicate specific restrictions or preferences then this should be explored with an experienced obstetrician, ideally in advance of labour.’ Our study suggests that very limited information is provided to expectant mothers about the possible use of instrumentation or about its possible consequences.

It is known that instrumentation increases the risk of a perineal tear. Furthermore, the risk of an obstetric anal sphincter injury in the UK is thought to be around 6% in primipara [15, 16]. Instrumentation is a well recognized risk factor for these injuries [15, 16]. The functional problems associated with an obstetric anal sphincter injury are well documented. These include anal incontinence, chronic pain, dyspareunia and evacuatory disorders. Such injuries are also associated with significant psychological morbidity and will have a negative impact on the quality of life of those affected.

It is also worth noting that obstetric forceps, known to be more damaging than ventouse deliveries, are not, or are very rarely, used in many other countries such as the USA and some countries in Europe.

Only 25% of the participants appeared to be able to describe the stages of labour. Those who replied they knew about stages of labour gave a partially correct answer or went off topic. Women at their first pregnancy appeared keener to get information from health professionals about what to expect for labour in comparison to women who had a previous delivery. Women who had two or more deliveries gave answers mainly based on their previous experience and not in line with formal information from health professionals.

Most participants did not attend antenatal classes because they were not offered any; some explained that this was related to the COVID‐19 outbreak. Some were offered some online classes.

Most of the women were not informed by the health professionals about the existence of instrumentation. The knowledge about its usage and risks is mainly related to personal experience, friends' experience or from self‐directed research on the internet.

Stohl [17], Trandel‐Korenchuk [18] and Moore [19] explained how important it is to explain to pregnant women extensively before childbirth the risks and complications related to use of instrumentation at childbirth. Similar results were found by Koster et al. [20], who looked at the perception of childbirth from women who had a traumatic childbirth. They found that women experienced a lack of information and consent from the clinicians at childbirth.

The first part of this study appears to demonstrate significant deficiencies in the awareness of the stages of labour, the risks of vaginal delivery and the use of instrumentation. When 14% of deliveries require instrumentation (the damaging consequences of such instrumentation are well recognized) and up to 6% may suffer an obstetric anal sphincter injury, it is difficult to believe that pregnant women are not better informed. It would be well outside acceptable practice to proceed with, for example, a right hemicolectomy without mentioning the risk of anastomotic leak (5%) or bile leak after cholecystectomy (1%). Yet, in this study, a failure to communicate the risk of perineal injury and instrumentation seemed to be common practice.

For the second part of this study, we found that there was no evidence of consent in 18.6% of the reviewed clinical notes. The nature of the emergency of the procedure still does not justify the absence of consent. ‘In the emergency situation, verbal consent should be obtained which should be witnessed by another care professional. Obstetricians and the witness to verbal consent must record the decision and the reason for proceeding to any emergency delivery without written consent’ [21].

Sturgeon et al. [22] noted that ‘NHS National Services Scotland (2018–2019) reported 123 medical negligence claims closed between January 2015 and December 2019 where the reason for claim was identified as “failure to obtain informed consent”.’ It is well recognized that informed consent is not simply a signature on a consent form. A signed consent form merely documents that the ‘process’ of informed consent has taken place [23].

Gerancher et al. [24] surveyed patients who were consented for epidural during labour and the use of a written consent associated with verbal consent gave the highest recall to participants about the consent process months after the childbirth. Only 20% of patients in our study had signed a consent form. Wada et al.'s [25] systematic review of studies focused on women's decision‐making about epidural analgesia for pain management in labour. They suggest that empirical evidence to date is insufficient to determine whether women undergoing labour have got full capacity when consenting to epidural analgesia. Given this uncertainty, sufficient information about pain management should be provided as part of antenatal education and consent should be taken for this prior to onset of labour. More prospective and retrospective studies are required on this topic.

One may rightly question the validity of consent for instrumentation taken during the second stage of labour. First, the patient may be under the effect of opiates, high doses of local anaesthetic or nitrous oxide, all with associated cognitive effects. Second, consent will often have been taken during a time of urgency, when maternal exhaustion and adverse fetal signs have become apparent. This may appear to add an element of coercion. All this would seem to be a long way from the NHS definition of informed consent ‘voluntary, informed, and with capacity’ (Consent to treatment—NHS, https://www.nhs.uk/conditions/consent‐to‐treatment/).

Furthermore, the justification that the use of instrumentation is an emergency life‐saving measure and that suboptimal consent may be tolerated should not be accepted. This is not a rare or unforeseen intervention. Remembering that one in seven patients require instrumentation, it is very much a foreseen intervention.

This study was limited by the fact that only a single institution was used and that the time frame for data collection was limited. The restrictions imposed due to the COVID‐19 pandemic are also likely to have had an impact on the ability to inform pregnant women about delivery, and this may have impacted the results reported in this study.

A multi‐centre study conducted along similar lines, over a longer period of time, may allow a better evaluation of the consent process in the antenatal and intrapartum periods across the UK.

## CONCLUSION

Further studies are needed to determine deficiencies in patient information about childbirth and consent for operative delivery.

## AUTHOR CONTRIBUTIONS

**Alessandra Orlando:** Conceptualization; investigation; writing – original draft; methodology; validation; visualization; writing – review and editing; software; formal analysis; project administration; data curation; resources. **Gregory P. Thomas:** Conceptualization; supervision; validation; writing – review and editing; visualization; project administration; resources. **Ruwan Fernando:** Conceptualization; supervision; project administration; writing – review and editing; validation; resources; visualization. **Jamie Murphy:** Conceptualization; methodology; validation; visualization; supervision; resources. **Nada Elsaid:** Project administration; data curation. **Stella Dilke:** Data curation; project administration. **Carolynne J. Vaizey:** Conceptualization; visualization; supervision; project administration; writing – review and editing; validation; resources.

## FUNDING INFORMATION

There has not been any funding for the studies.

## CONFLICT OF INTEREST STATEMENT

No conflict of interest identified.

## ETHICS STATEMENT

Ethical approval was obtained from the West Midlands South Birmingham Research Ethics Committee (Iras number 289693) in December 2020.

## Data Availability

The data that support the findings of this study are available on request from the corresponding author. The data are not publicly available due to privacy or ethical restrictions.
